# P-57. Durability of *SARS-CoV-2* specific Cellular Immune Response after COVID-19 Vaccination in PLWH

**DOI:** 10.1093/ofid/ofae631.264

**Published:** 2025-01-29

**Authors:** Amit Sehgal, Neeraj Nischal, Sanjay Ranjan, Niranjan Mahishi, Pankaj Jorwal, Naveet Wig, Pragati Grover, Maroof a Khan, Amit Awasthi, Akshay Binayke

**Affiliations:** All India Institute of Medical Sciences, New Delhi, NEW DELHI, Delhi, India; All India Institute of Medical Sciences, New Delhi, NEW DELHI, Delhi, India; ALL INDIA INSTITUTE OF MEDICAL SCIENCES NEW DELHI, DELHI, Delhi, India; ALL INDIA INSTITUTE OF MEDICAL SCIENCES NEW DELHI, DELHI, Delhi, India; All India Institute of Medical Sciences, New Delhi, Delhi, India; All India Institute of Medical Sciences, New Delhi, Delhi, India; ALL INDIA INSTITUTE OF MEDICAL SCIENCES NEW DELHI, DELHI, Delhi, India; ALL INDIA INSTITUTE OF MEDICAL SCIENCES NEW DELHI, DELHI, Delhi, India; Translational Health Science and Technology Institute, HARYANA, Haryana, India; Translational Health Science and Technology Institute, HARYANA, Haryana, India

## Abstract

**Background:**

People living with HIV (PLWH) face the dual challenge of human immunodeficiency virus(HIV) infection and severe acute respiratory syndrome coronavirus-2 (SARS-CoV-2) infection. It is associated with severe outcomes and a higher risk of mortality.

In January 2021, the vaccination campaign against SARS-CoV-2 was launched in India and started mass administration of two types of vaccines. The two vaccines used on a large scale were, a recombinant, replication-deficient chimpanzee adenovirus vectored vaccine/ ChAdOx1 nCoV-19 (Covishield) and whole virion inactivated vaccine/ BBV152 (Covaxin).The study of cellular immune responses to COVID-19 vaccination in PLWH was vital for safeguarding this vulnerable population.

**Figure:**
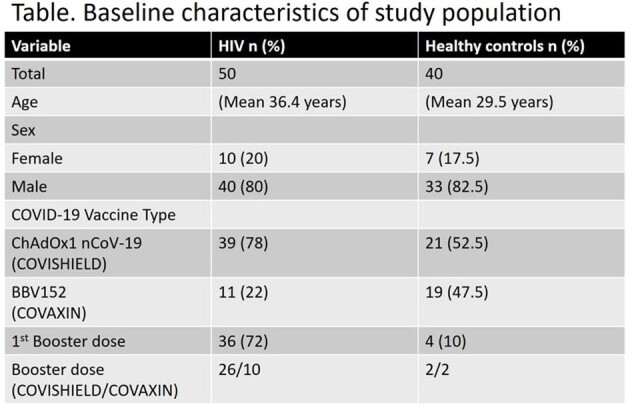

**Methods:**

Study design: Prospective observational study

Study population: The individuals recruited in the study were vaccinated with at least 2 doses and/or booster dose of the COVID-19 vaccine (COVISHIELD/COVAXIN). The individuals were segregated into two groups (PLWH and non-HIV-infected healthy individuals).

Testing Method: Peripheral blood mononuclear cell (PBMC) were cultured to test the SARS-CoV-2-specific T cell immune response using activation induced marker/ intracellular cytokine staining (AIM/ICS) assay.

Distribution of CD4 T lymphocytes count in HIV patients with mean CD4 count 470 cell/µl.

**Figure:**
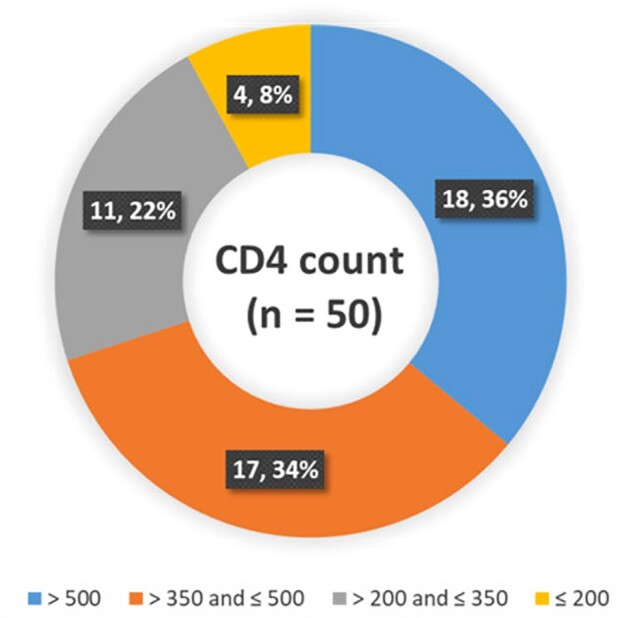

**Results:**

We conducted did this study in two phases. The first phase was conducted between August 2022 and December 2022, while the second phase, or the follow-up, took place from January 2023 to December 2023. In the first phase/ first visit (V1), we recruited 50 adult PLWH and 40 adult healthy controls. Subsequently, in phase 2/follow-up/ second visit (V2), we achieved a follow-up rate of 100% in each arm.

*SARS-CoV2-*specific cellular immune response after COVID-19 vaccination was comparable and durable IN PLWH as compared to healthy controls.

**Figure:**
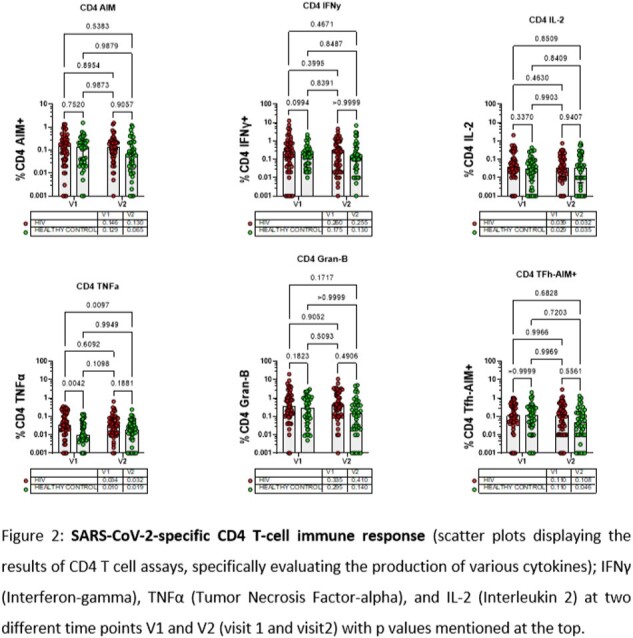

**Conclusion:**

Comparable and durable immune response was observed in PLWH population on regular highly active antiretroviral therapy (HAART), with stable viral suppression and good CD4+ T cell counts. This was consistent over time across 1 year gap in both phases of the study and across different vaccine types. This outcome indicates that the cellular immune responses in our PLWH cohort is comparable and durable to that of the general population, which is a reassuring finding for the public health.

SARS-CoV-2-specific CD8 T-cell immune response (scatter plots displaying the results of CD8 T cell assays, specifically evaluating the production of various cytokines (IFNγ (Interferon-gamma), TNFα (Tumor Necrosis Factor-alpha), and IL-2 (Interleukin 2), Granzyme B (Gran-B) at two different time points V1 and V2 (visit 1 and visit 2) with p values mentioned at the top.

**Figure:**
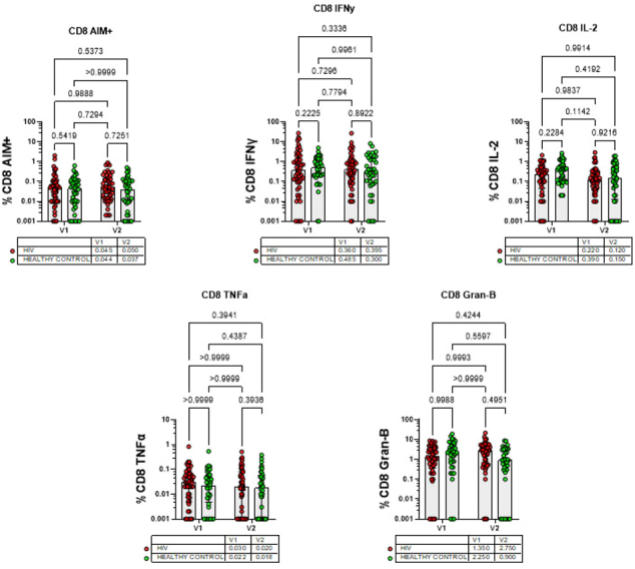

**Disclosures:**

**All Authors**: No reported disclosures

